# Rare Case of Olmesartan Induced Enteropathy

**DOI:** 10.51894/001c.6383

**Published:** 2017-12-19

**Authors:** Richa Handa, Akhil Rahman, Vivek Kak

**Affiliations:** 1 Henry Ford Allegiance Health, Internal Medicine, PGY3 Resident, Jackson, MI; 2 Henry Ford Allegiance Health, Internal Medicine, Attending Physician, Jackson, MI; 3 Henry Ford Allegiance Health, Internal Medicine, Program Director, Jackson, MI

**Keywords:** endoscopy, diarrhea, enteropathy, olmesartan

## Abstract

Olmesartan (brand name Benicar) is an antihypertensive drug clinicians commonly use to treat high blood pressure. Olmesartan induced enteropathy (OSE) is a rare entity that authors first identified in 2012. The etiological basis of OSE remains unclear, although authors have suggested that this condition could be due to alternations in cell mediated immune responses induced by the drug. The objective of the case report is to describe a patient who presented with diarrhea and was eventually diagnosed with OSE. A female patient in her later 60s presented to an emergency room after two recent hospitalizations with profound diarrhea, generalized weakness and weight loss. She underwent a diagnostic workup including endoscopy and colonoscopy. The patient’s endoscopy with duodenal biopsy revealed villous atrophy with attenuated and blunted villi with intraepithelial CD3 positive T lymphocytes, suggestive of gluten-induced enteropathy. When the patient’s symptoms did not improve after the authors placed her on a gluten free diet for a few days, they further investigated her for secretory diarrhea, including Gastrin, Somatostatin and Vasoactive Intestinal Peptide lab values that they found to be within normal limits. Due to the patient’s lack of improvement with initial treatment, the authors suspected OSE and stopped her olmesartan and the patients’ symptoms gradually improved in three weeks.

## INTRODUCTION

Olmesartan is an Angiotensin II receptor blocker (ARB) approved in 2002 for treatment of hypertension. In 2012, authors first described a sprue-like enteropathy associated with olmesartan in the literature, with more cases since reported.^1–3^ This led the FDA to categorize olmesartan-induced enteropathy (OSE) as a drug-induced adverse reaction in 2013.^4^ However, knowledge concerning this condition is still not widespread in the clinical setting. Early diagnosis and discontinuation of olmesartan can help in preventing severe and life-threatening conditions.^2,4^ It is an immune-mediated entity found to be associated with a past history of autoimmunity, presence of anti-nuclear antibodies, positivity of DQ2 and DQ8 haplotypes and presence of polyclonal intraepithelial lymphocytes.

The mechanism of OSE is still unknown. It has been postulated that villous atrophy has been caused by AT2 receptors activated by Angiotensin II. Treatment primarily includes discontinuation of the medication as well as monitoring the patient for three to four weeks for improvement in symptoms. In this paper, we present a case of OSE in which a patient presented with severe diarrhea, generalized weakness and weight loss.

### Case Report

A female in her later 60s with a history of hypertension on amlodipine and olmesartan presented to an emergency room with complaints of profound diarrhea and generalized weakness that had started a month ago along with a twenty-pound weight loss. Her recent medical history was notable for two recent hospital admissions with similar complaints and an extensive workup for diarrhea, including an endoscopy and a colonoscopy, which were negative. The patient had not recently travelled out of her mid-Michigan town.

Stool studies for infectious etiology were negative. During this visit to the emergency room, providers found her to be hypotensive with a blood pressure of 70/50 Mm Hg and heart rate of 120, and extremely lethargic. The patient’s physical examination was remarkable for dry mucous membrane, although there were no other focal findings. Her initial lab tests revealed a renal insufficiency with a Creatinine of 3.5 mg/dl, which improved with aggressive hydration.

**Image 1:**
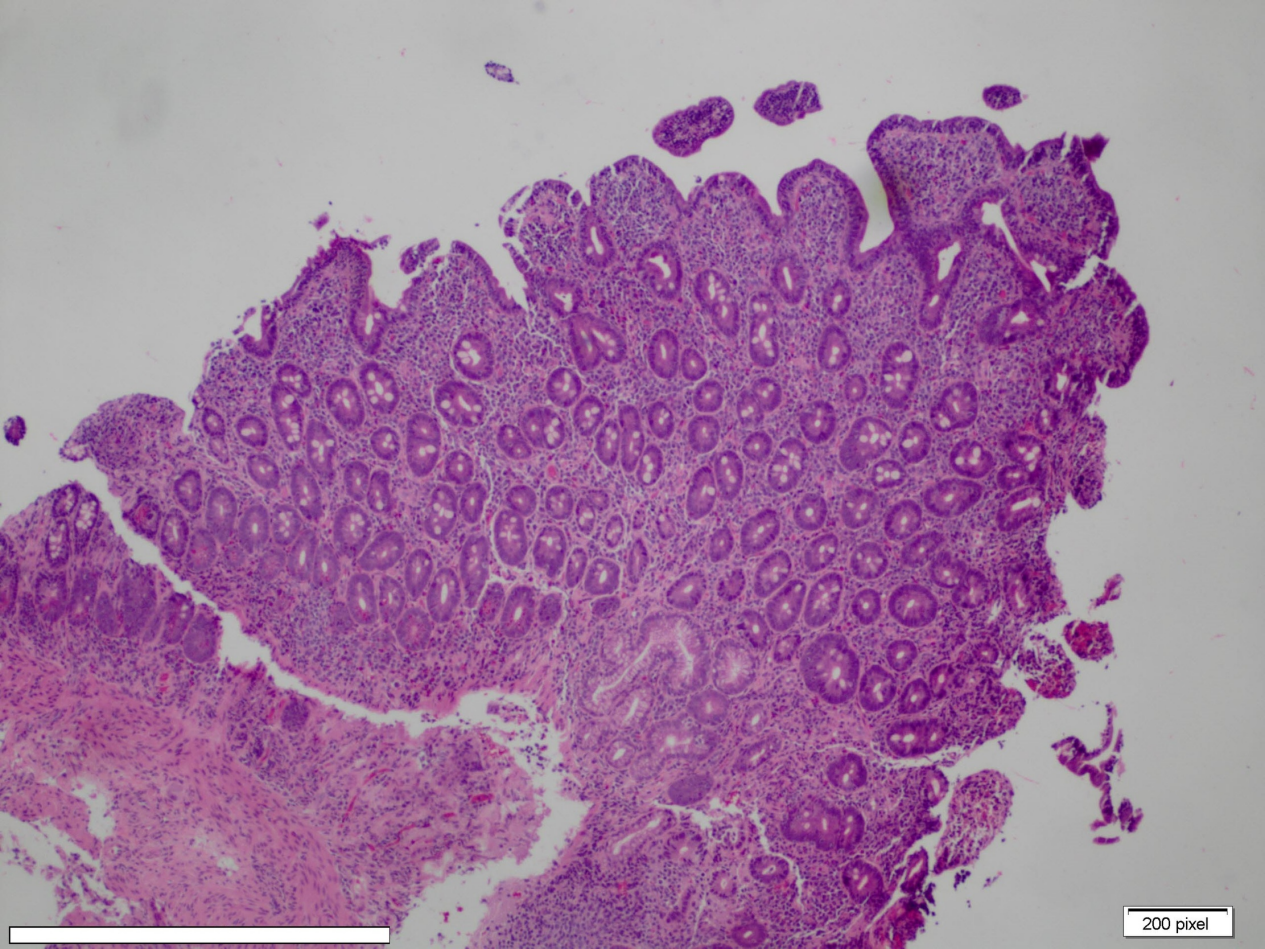
Duodenum Biopsy (40x) showing Partial Villous Atrophy (Attenuated/Blunted Villi) and Increased Intraepithelial Lymphocytes.

**Image 2:**
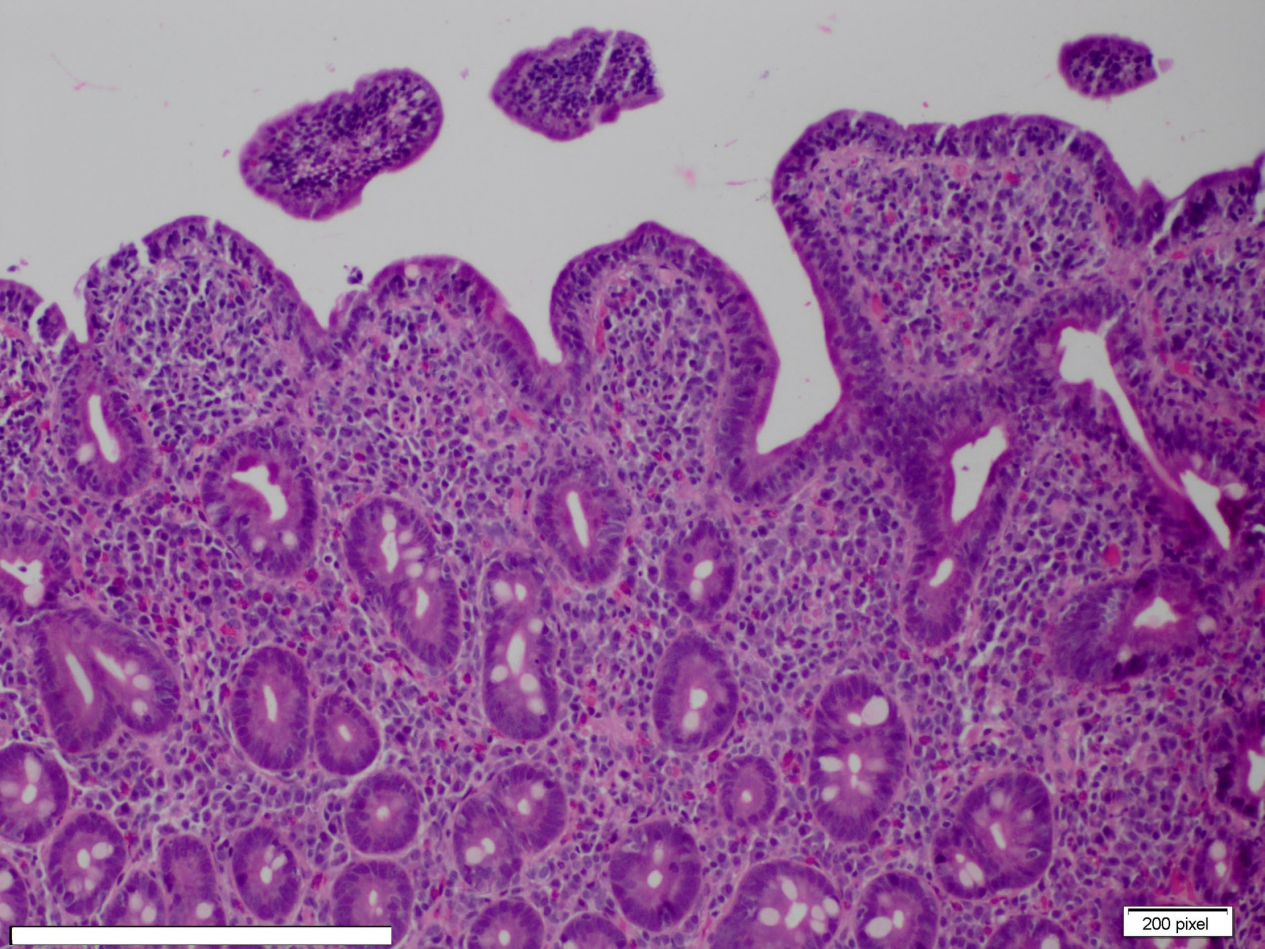
Duodenum Biopsy (100x) showing Partial Villous Atrophy (Attenuated/Blunted Villi) and Increased Intraepithelial Lymphocytes.

Her stool studies for celiac disease serology were also negative. She had a borderline positive ANA test at a titer of 1:160 with a homogeneous pattern. Homogenous pattern is a diffuse type of fluorescent pattern seen on indirect fluorescent antibody test. After two days in the hospital, she had a repeat endoscopy with duodenal biopsy, which revealed villous atrophy with attenuated and blunted villi with intraepithelial CD3 positive T lymphocytes (Images 1 and 2), suggestive of gluten enteropathy.

She was placed on gluten free diet for four weeks without a significant improvement in her symptoms. She was further investigated for secretory diarrhea, including Gastrin, Somatostatin and Vasoactive Intestinal Peptide laboratory markers that were each within normal limits. Due of her lack of improvement, the authors started to suspect OSE. Once olmesartan was stopped, the patients’ symptoms gradually improved within three weeks.

## DISCUSSION

Olmesartan induced enteropathy is likely an immune-mediated condition that has been associated with a history of autoimmunity, presence of anti-nuclear antibodies, and presence of polyclonal intraepithelial lymphocytes.^5^ The histology associated with OSE is characterized by villous atrophy and increase in intra-epithelial lymphocytes such as CD3 and CD8 in the small bowel. Authors have also found this histology in other disorders such as Crohn's disease, enteric infections, collagenous sprue, tropical sprue, common variable immunodeficiency, hematological malignancies and autoimmune enteropathy.^1,2^

Various medications such as neomycin, sulindac, colchicine and immune-suppressants such as azathioprine, mycophenolate mofetil, methotrexate, vincristine and ipilimumab can cause similar histological changes in small bowel.^6–10^ The mechanism of these changes in OSE is still unknown but it has been postulated that villous atrophy in this condition is caused by AT2 receptors activated by Angiotensin II.^11^ Angiotensin II binds to two receptor forms, AT1 and AT2. AT1 receptor activates growth-promoting factors whereas AT2 receptors cause apoptosis of enterocytes by causing upregulation of pro- apoptotic proteins such as Bax and GATA-6 and by inhibiting anti pro-apoptotic proteins such as Bcl-2.^12,13^

Recently, it has also been shown that drugs that inhibit AT1 receptors can cause translocation of AT 2 receptors from cytosol to the external membrane in the presence of high concentrations of Angiotensin II.^14^ Olmesartan has been shown to have a high affinity for AT1 receptors, but when AT1 receptors are saturated with olmesartan, then Angiotensin II starts binding to AT2 receptors resulting in apoptosis of enterocytes and villous atrophy.^11^ According to another hypothesis, increased bacterial counts were reported which was possibly secondary to alteration in luminal microbiome, but anti-bacterial medications were not found to be helpful.^10^ In addition, one literature review found positivity of DQ2 and DQ8 haplotypes and absence of celiac disease serologies in patients with OSE.^11^

There are various clinical features of OSE. In one systematic literature review, 54 patients were studied and according to their results, nausea and vomiting were present in 45% of cases, fatigue in 56% of cases, diarrhea in 95% of cases, weight loss in 89% of cases, abdominal pain in 37% and bloating in 29% of cases.^11^ The mean age of onset of symptoms was 69 years with age range of 47 years to 87 years,^11^ and the mean duration of olmesartan therapy before patients developed symptoms in several studies was found to be between six months and seven years.^2,11,15–18^ The most common laboratory findings found to be associated with OSE are normochromic normocytic anemia and hypoalbunemia.^11^

Endoscopic examination with high definition scopes usually shows marked villous atrophy and flattening of duodenal villi.^11^ Other endoscopic findings found in OSE are nodularity in duodenal bulb ^16^ and duodenal ulcers,^17^ although normal duodenal patterns can also be found.^18^ Histopathologically flattening of villous pattern is the most common finding observed in various studies.^1,2^

Also, the most common misdiagnosis in patients with these findings is celiac disease, with patients not improving after following a gluten free diet.^1,19,20^ The absence of two main histopathological findings that can sometimes be helpful to distinguish OSE from celiac disease are the absence of duodenal intraepithelial lymphocytes and a thickened sub epithelial collagen band.^21^

In one systemic literature review, only 65% and 33% of patients with villous atrophy diagnosed histopathologically had increased duodenal intraepithelial lymphocytes and sub epithelial collagen band respectively, though large studies are still needed to describe this association.^11^ Also, improvement in symptoms, clinical remission and return of duodenal mucosa to its normal architecture after discontinuation of olmesartan have been found in various case studies of olmesartan induced enteropathy.^2,5^

The proposed reasons for unusual enteropathy associated with olmesartan as compared to other ARB antihypertensive medications include different pharmacokinetic properties.^10^ Two other ARBs that were rarely found be associated with enteropathy are valsartan ^22^ and irbesartan.^23^

Recently, skin lesions associated with OSE have also been described.^24^ These lesions appear simultaneously with digestive symptoms of OSE and also regress completely after discontinuation of olmesartan.^24^ Certainly, more case reports, case series and studies are required to confirm the association of these skin lesions with olmesartan.^24^ In order to differentiate these lesions from dermatitis herpetiformis associated with celiac disease, biopsy of lesions should be done.^24^ According to one case report, the biopsy in OSE showed pemphigoid or acquired bullous epidermolysis-like histological findings.^24^

## CONCLUSIONS

Olmesartan induced enteropathy is an immune-mediated entity and the histology associated with this condition is characterized by villous atrophy and increase in intra-epithelial lymphocytes such as CD3 and CD8 in the small bowel. In this case study, endoscopy with duodenal biopsy also revealed villous atrophy with attenuated and blunted villi with intraepithelial T lymphocytes positive for CD3 cells. Other endoscopic findings found in OSE are nodularity in duodenal bulb and duodenal ulcers. In some cases, normal duodenal patterns have also been found with symptoms including diarrhea, generalized weakness and weight loss.^18^

Also, the most common misdiagnosis in patients with these findings is celiac disease, though OSE patients have not shown improvement after following a gluten free diet. Recently, skin lesions have also been found to be associated with OSE.^24^ The treatment includes stoppage of olmesartan. OSE should be suspected in patients with these types of symptoms that remain unexplained by other common causes of enteropathy such as celiac disease and other autoimmune enteropathies.

### Conflict of Interest

The authors declare no conflict of interest.
